# Digital mental health in China: a systematic review

**DOI:** 10.1017/S0033291721003731

**Published:** 2021-11

**Authors:** Xiaolong Zhang, Shôn Lewis, Joseph Firth, Xu Chen, Sandra Bucci

**Affiliations:** 1 Division of Psychology and Mental Health, School of Health Sciences, Faculty of Biology, Medicine and Health, Manchester Academic Health Science Centre, The University of Manchester, Manchester, UK; 2 The National Clinical Research Center for Mental Disorders & Beijing Key Laboratory of Mental Disorders, Beijing Anding Hospital, Capital Medical University, Beijing, China; 3 Greater Manchester Mental Health NHS Foundation Trust, Manchester, UK; 4 NICM Health Research Institute, Western Sydney University, Westmead, Australia; 5 Advanced Innovation Center for Human Brain Protection, Capital Medical University, Beijing, China

**Keywords:** China, co-production, cultural adapation, digital mental health, schizophrenia, systematic review

## Abstract

Mental health problems are highly prevalent in China; however, China's mental health services lack resources to deliver high-quality care to people in need. Digital mental health is a promising solution to this short-fall in view of the population's digital literacy. In this review, we aim to: (i) investigate the effectiveness, acceptability, usability, and safety of digital health technologies (DHTs) for people with mental health problems in China; (ii) critically appraise the literature; and (iii) make recommendations for future research directions. The databases MEDLINE, PsycINFO, EMBASE, Web of Science, CNKI, WANFANG, and VIP were systemically searched for English and Chinese language articles evaluating DHTs for people with mental health problems in mainland China. Eligible studies were systematically reviewed. The heterogeneity of studies included precluded a meta-analysis. In total, 39 articles were retrieved, reporting on 32 DHTs for various mental health problems. Compared with the digital mental health field in the West, the Chinese studies targeted schizophrenia and substance use disorder more often and investigated social anxiety mediated by shame and culturally specific variants, DHTs were rarely developed in a co-production approach, and methodology quality was less rigorous. To our knowledge, this is the first systematic review focused on digital mental health in the Chinese context including studies published in both English and the Chinese language. DHTs were acceptable and usable among Chinese people with mental health problems in general, similar to findings from the West. Due to heterogeneity across studies and a paucity of robust control trial research, conclusions about the efficacy of DHTs are lacking.

## Introduction

Mental health problems are a major cause of global disease burden (Vigo, Thornicroft, & Atun, 2016), and constitute the largest single source of world economic burden, greater than many physical health diseases (Mental Health Foundation, 2015). Mental health problems are prevalent in China; the 12-month prevalence rate of any mental health problem is 9.3%, and lifetime prevalence is 16.6% (Huang et al., 2019). Despite the tremendous demands on mental healthcare, there is a vast shortage of resources to deliver high-quality care to help-seeking individuals across China. The scarcity of mental health professionals in China is significant, with an estimated shortage of 40 000 psychiatrists relative to the population need (Wu, Zhao, & Ye, 2016). Since the Chinese healthcare system is designed to be delivered in tertiary hospitals (Yip & Hsiao, 2014), well-trained mental health professionals are concentrated in psychiatric hospitals in urban areas (Liu et al., 2013), leaving a significant short-fall in accessing mental healthcare for rural/remote communities (Xiang, Ng, Yu, & Wang, 2018). A national programme aimed at integrating hospital and community-based mental health services for people with severe mental health problems (i.e. the 686 programme) has more than 6 million current enrollees with severe mental illness, and more than 70% of the enrollees have received basic medication treatment (Lu, 2019; Ma, 2012). However, further mental health service reform is indispensable to close the treatment gap and promote mental health in China (Liang, Mays, & Hwang, 2018b; Que, Lu, & Shi, 2019).

With the Chinese economy booming and the fast-paced development of digital health technologies (DHTs), digital mental health is a promising solution to this short-fall. China's use of digital technologies has grown exponentially in the past decade, with ownership rates of smartphone in China is now 96% (Chou, Chung, & Lam, 2019), which comparable to Western countries. In a recent review (Yin et al., 2020), 172 mental health smartphone apps have been developed in Chinese and can be downloaded from app stores. Since coronavirus disease 2019 (COVID-19), online mental health services have also significantly increased in China (Liu et al., 2020), as has been seen in many other countries worldwide (Torous, Myrick, Rauseo-Ricupero, & Firth, 2020). Although four systematic reviews on digital mental health in low- and middle-income countries (LMICs; Carter, Araya, Anjur, Deng, & Naslund, 2021; Fu, Burger, Arjadi, & Bockting, 2020; Naslund *et al*. 2017) or resource-limited settings (Kaonga & Morgan, 2019) have been conducted, findings have been interpreted in the general context of LMICs. However, these reviews excluded a number of studies published in Chinese/Chinese-based journals, and to date, there have been no systematic reviews examining digital mental health in a specific Chinese context. Therefore, in order to understand how digital mental health can help solve the mental health crisis for the most populated country and the second largest economy in the world, and to inform the development of DHTs in other LMICs, a systematic review of DHTs in the Chinese context is timely. The aims of the current systematic review are to: (i) investigate the effectiveness, acceptability, usability, and safety of digital mental health interventions for people with mental health problems in the Chinese context; (ii) critically appraise the literature; and (iii) make recommendations for future research directions for developing digital mental health in China.

## Method

### Search strategy

This study was conducted and reported in accordance with the Preferred Reporting Items for Systematic Review and Meta-Analyses (PRISMA) guidelines (Moher, Liberati, Tetzlaff, Altman, & Group, 2009). A protocol of the review was registered with PROSPERO (CRD42020192185). Four English language databases (MEDLINE, PsycINFO, EMBASE via Ovid, and Web of Science), and three Chinese language databases (China National Knowledge of Infrastructure, WANFANG Data, and VIP Information) were systemically searched. Terms were used to search title, abstract, keywords, and subject headings. A detailed search strategy in both English and Chinese is provided in online Supplementary Table S1. Results were limited to English and Chinese language peer-reviewed journal publications. A hand search of reference lists of eligible papers was conducted to identify additional relevant studies. Two reviewers (XZ and XC) independently screened 100% of the papers returned from the search in both English and Chinese at each of the title, abstract, and full-text paper levels, and assessed study eligibility with queries resolved through discussion with the research team. Primary searches were completed in February 2020 and updated in June 2021.

### Eligibility criteria

Eligible studies were peer-reviewed, quantitative, and qualitative studies aimed at evaluating effectiveness, acceptability, usability, or safety of digital mental health interventions. Study designs included randomised controlled trials (RCTs), non-randomised controlled trials (non-RCTs), case report/case-series studies, survey studies, interview, and focus group studies. We included studies conducted in mainland China with participants who met DSM or ICD criteria where diagnosis was operationalised as those who met clinical threshold assessed by a validated clinical tool or confirmed by a treating clinician, with no restriction on age. Studies conducted in Hong Kong or Taiwan were not included, in view of the different healthcare contexts. Studies focused on DHTs targeting mental health problems, including short message service (SMS), mobile apps, computer programmes, software, interactive websites, social media, wearable and ambient sensors, virtual reality, or artificial intelligence. Programmes that focused solely on information provision (e.g. internet-based psychoeducation with no additional interactive function), or purely provided electronic communication (e.g. contact via telephone, videoconference, text, or email with professional without any therapeutic content) were excluded. Studies where a mental health problem was not the primary focus of the paper, such as physical health problems, health behaviours (e.g. smoking cessation), neurodegenerative disorders (e.g. dementia), and internet addiction, were excluded.

### Data extraction and analytic plan

**Fig. 1.**
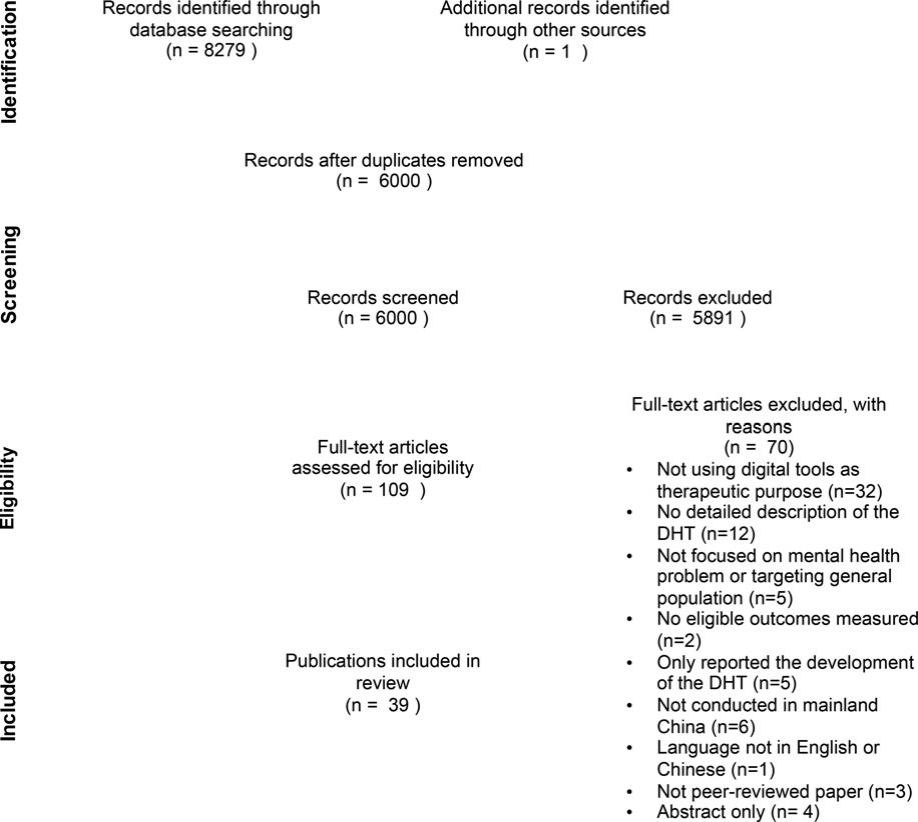
PRISMA flow chart of study selection.

The study selection process is summarised in Fig. 1. Cohen's kappa was used to assess interrater reliability, and strong interrater agreements were found for both the title and abstract level (*κ* = 0.89) and the full text level (*κ* = 0.94) screening. The following information was extracted from selected papers: (a) general information (author, article title, type of publication, country of origin); (b) study characteristics (study aim/objectives, study design, study inclusion and exclusion criteria, recruitment procedures used); (c) participant characteristics (age, gender, ethnicity, diagnostic information, number of participants in each characteristic category for intervention and control groups); (d) study setting (country, type of service); (e) intervention (intervention name, length, frequency, intervention development, summary of intervention, theoretical basis); and (f) outcome data/results (unit of assessment/analysis statistical techniques used). Quantitative and qualitative data were integrated using a narrative synthesis approach. Due to the heterogeneity regarding study designs and outcomes, a meta-analysis was not conducted.

### Quality assessment

Due to the diversity of study designs included, the Mixed Methods Appraisal Tool (MMAT; Hong et al., 2018) was used to assess the quality of studies. The MMAT permits the appraisal of the methodological quality of studies across five categories, including qualitative research, RCTs, non-RCTs, quantitative descriptive studies, and mixed-methods studies. The quality assessment was independently carried out by two reviewers (XZ and XC; *κ* = 0.97). Discrepancies were due to some studies not explicitly describing their methodology; therefore, further discussion was required before consensus was reached. Criteria from the MMAT are shown in online Supplementary Table S2.

## Results

The database search returned 8279 results. Duplicate results were removed (*n* = 2280 articles) using reference manager software before titles and abstracts were screened for inclusion (*n* = 5999 articles). A further 5891 papers were excluded by title and abstract screening. Full-text papers were assessed for the remaining 108 articles; 70 were excluded (reasons are shown in Fig. 1). One further article was identified following a hand search of reference lists of eligible studies. Finally, 39 articles were retrieved in total. The process of study selection is demonstrated in Fig. 1.

### Characteristics of included studies

A summary of the characteristics of selected studies is shown in Table 1. A more detailed description of each study is provided in online Supplementary Table S3. Included studies were published from 2010 to 2021. Eleven papers were published in Chinese; 28 papers were published in English. There were 27 RCTs, four non-RCTs, five case-series studies, and three qualitative studies.

**Table 1.** Characteristics of included studies (organised by type of digital health technologies)

Author, location	Study design, sample	Study aim and summary of intervention (description, intensity, PPI)	Outcome measures	Key findings	Quality rating
*Computerised programme*					
Tan et al. (2019), mainland China	RCT; schizophrenia; *N* = 311 (IG = 196, CG = 115)	**Study aim:** To investigate the efficacy of a CCRT for improving neurocognitive functions in schizophrenia. **Intensity:** 12-weeks, 4–5 sessions per week (45 min per session, 50 sessions in total). **PPI:** No.	**Cognitive outcomes:** MCCB, Wechsler Adult Intelligence Scale-III Digit Span Test, WCST **Functional outcomes:** UPSA, NOSIE-30, Rosenberg Self-Esteem Scale **Symptoms:** PANSS	**Cognitive functions:** A significant benefit MCCB total score for CCRT (*F* = 5.62, *p* = 0.02). **Functional outcomes:** No significant effect on functional outcomes for CCRT. However, a significant decrease on NOSIE negative factors for the active control group at follow-up, with no change in the CCRT group (*F* = 4.21, *p* = 0.04). **Symptoms:** A significant effect on the PANSS negative symptom score for CCRT (*F* = 4.82, *p* = 0.03).	2.1 = 1 2.2 = 1 2.3 = 1 2.4 = 1 2.5 = 1
Zhu et al. (2020b, 2020c), mainland China	RCT; schizophrenia; *N* = 157 (IG = 78, CG = 79)	**Study aim:** To explore the effects of CCRT on cognitive functioning and social functioning in schizophrenia. **Intensity:** 12-weeks, 4–5 sessions per week. **PPI:** No.	**Cognitive outcomes:** MCCB total score and domain score **Functional outcomes:** PSP, UPSA **Symptoms:** PANSS	**Cognitive outcomes:** Relative to control group, a significant improvement in the MCCB total score ES = 0.31 (0.01–0.62) and in social cognition ES = 0.35 (0.01–0.69) was found in intervention group. **Functional outcomes:** A significant improvement on PSP (ES = 0.44 (0.13–0.75)) was found in intervention group. No between group differences on UPSA. **Symptoms:** No between group differences on PANSS.	2.1 = 2 2.2 = 1 2.3 = 1 2.4 = 1 2.5 = 1
Liao et al. (2016), mainland China	RCT; schizophrenia; *N* = 59 (IG = 31, CG = 28)	**Study aim:** To evaluate efficacy of a computerised cognitive remediation therapy to adult patients with schizophrenia. **Intensity:** 30-days, 1 session per day (25–40 min per session, 30 sessions in total). **PPI:** No.	**Cognitive outcomes:** ACCT **Symptoms:** BPRS, CGI-S	**Cognitive outcomes:** A significant improvement on digit sequence test (*t* = 2.09, *p* = 0.04) and Stroop test (*t* = 2.64, *p* = 0.01) of the ACCT in treatment group *v*. control group. **Symptoms:** No significant effect on symptoms.	2.1 = 1 2.2 = 1 2.3 = 1 2.4 = 2 2.5 = 1
Byrne et al. (2015), mainland China	Non-RCT; schizophrenia; *N* = 40 (IG = 20, CG = 20)	**Study aim:** To implement and evaluate a brief computerised cognitive remediation programme designed to improve memory, attention, and facial affect recognition in outpatients with chronic schizophrenia. **Intensity:** 6-weeks, complete at least 12 sessions. **PPI:** No.	**Symptoms:** PANSS **Cognitive outcomes:** the Hong Kong List Learning Test and the Letter-Number Sequencing Task **Functional outcomes:** PSP, facial affect recognition	**Symptoms:** No significant between group differences in clinical symptoms. Significant improvements on PANSS positive symptoms (*t* = 2.26, *p* = 0.036), negative symptoms (*t* = 2.52, *p* = 0.021), and general psychopathology (*t* = 3.26, *p* = 0.004) in intervention group at post-treatment. **Cognitive outcomes:** No significant between group differences in cognitive outcomes. **Functional outcomes:** A significantly greater improvement on the PSP in the intervention group *v*. the control group (*F* = 1.85, *p* = 0.018). The pre-post change in the total facial recognition score in the intervention group was significant (*t* = −2.60, *p* = 0.018), but with no significant between group differences.	3.1 = 0 3.2 = 1 3.3 = 1 3.4 = 1 3.5 = 1
Byrne et al. (2013), mainland China	RCT; schizophrenia; *N* = 51 (IG = 24, CG = 27)	**Study aim:** To develop and evaluate a computerised cognitive remediation programme designed to improve memory and attention for people with schizophrenia. **Intensity:** 6-weeks, complete at least 12 sessions. **PPI:** No.	**Symptoms:** PANSS, CGI **Social functioning:** PSP **Cognitive function:** HKLLT, LNST	**Symptoms:** Significant improvements on PANSS positive symptom (*F* = 7.36, *p* = 0.011) and negative symptom (*F* = 21.14, *p* = 0.000), and CGI (*F* = 8.37, *p* = 0.007) in the intervention group *v*. control group. **Social functioning:** A significant improvement on PSP (*F* = 14.04, *p* = 0.001) in the intervention group *v*. control group. **Cognitive function:** The intervention group also performed better than the control group on the post-treatment measure on LNST (*F* = 11.41, *p* = 0.002), but not on HKLLT.	2.1 = 1 2.2 = 0 2.3 = 0 2.4 = 1 2.5 = 0
Hu et al. (2021), mainland China	RCT; schizophrenia; *N* = 162 (IG = 81, CG = 81)	**Study aim:** To explore the effects of computerised cognitive remediation therapy on cognitive function in schizophrenia. **Intensity:** 12-weeks, 4 sessions per week, each session lasts 45 min. **PPI:** No.	**Cognitive outcomes:** RBANS	**Cognitive outcomes:** A significant improvement on RBANS (*p* < 0.001) in treatment group *v*. control group.	2.1 = 1 2.2 = 1 2.3 = 1 2.4 = 1 2.5 = 2
Zhu *et al*. (2018a), mainland China	RCT; schizophrenia; *N* = 86 (IG = 43, CG = 43)	**Study aim:** To investigate the effects of computerised cognitive remediation therapy on cognitive function, levels of self-esteem and social performance in schizophrenia. **Intensity:** 8-weeks, 1 session per day, each session lasts 20 min. **PPI:** No.	**Cognitive function:** WCST, TMT, and digit span and coding subtests of the WAIS-RC **Self-esteem:** SES **Social functioning:** PSP	**Cognitive outcomes:** A significant improvement on TMT and digit span subtest of the WAIS-RC (*p* < 0.05) in treatment group *v*. control group posttreatment. **Self-esteem:** A significantly higher SES score (*p* < 0.05) in treatment group *v*. control group. **Social functioning:** A significantly higher PSP score (*p* < 0.05) in treatment group *v*. control group.	2.1 = 1 2.2 = 1 2.3 = 2 2.4 = 2 2.5 = 2
Zhang et al. (2016b), mainland China	RCT; schizophrenia; *N* = 80 (IG = 40, CG = 40)	**Study aim:** To explore the effects of computerised cognitive remediation therapy on cognitive function in schizophrenia. **Intensity:** 6-weeks, 3 sessions per week, each session last 45 min. **PPI:** No.	**Cognitive outcomes:** MCCB total score and domain score **Symptoms:** PANSS	**Cognitive outcomes:** A significant improvement on working memory of the MCCB (*p* < 0.05) in treatment group *v*. control group posttreatment. **Symptoms:** No significant between group differences in clinical symptoms.	2.1 = 1 2.2 = 1 2.3 = 1 2.4 = 2 2.5 = 1
Zhu *et al*. (2020a), mainland China	RCT; MDD; *N* = 67 (IG = 35, CG = 32)	**Study aim:** To explore the effects of computerised cognitive remediation therapy on the improvement of cognitive functioning in patients with MDD. **Intensity:** 8-weeks, 1 session per day, each session lasts 20 min. **PPI:** No.	**Cognitive outcomes:** WCST, TMT, Stroop test	**Cognitive outcomes:** A significant improvement on WSCT and TMT-B (*p* < 0.05) in treatment group *v*. control group posttreatment.	2.1 = 1 2.2 = 1 2.3 = 2 2.4 = 2 2.5 = 2
Sit et al. (2020), mainland China	Non-RCT; depression; *N* = 51 (IG = 30, CG = 21)	**Study aim:** To obtain preliminary evidence for the effects of the computerised positive mental imagery training on depression. **Intensity:** 4-weeks, 2 sessions per week, each session lasts 30 min. **PPI:** No.	**Symptoms:** BDI-II **Psychological outcomes:** PANAS, RRS	A significant group × time interaction was found on depressive symptoms, positive and negative affect, and rumination (*F*_(8,150)_ = 2.19, *p* = 0.031), with better outcomes in intervention group compared to control group.	3.1 = 1 3.2 = 1 3.3 = 1 3.4 = 1 3.5 = 1
*Internet-based programme*					
Lin et al. (2020), mainland China	RCT; anxiety; *N* = 80 (IG = 55, CG = 25)	**Study aim:** To investigate the efficacy of ICBT for individuals with social anxiety and different levels of Taijin Kyofusho^b^ symptoms in China. **Intensity:** 8-weeks, 1 module each week. **PPI:** No.	**Symptoms:** SIAS, SPS, TKSS	Among participants in the treatment group, 42% and 29% reported significant post-treatment reductions in SIAS and on SPS, respectively. Participants in the treatment group with higher pre-treatment Taijin Kyofusho levels showed significantly greater reductions in TKSS scores, with an improvement rate of 44%.	2.1 = 1 2.2 = 0 2.3 = 0 2.4 = 0 2.5 = 0
Wang et al. (2020), mainland China	RCT; anxiety; *N* = 210 (IG1 = 70, IG2 = 70, IG3 = 70)	**Study aim:** To investigate the effectiveness of ICBT on reducing shame proneness, and which modules of the ICBT contributed to the reduction for participants with SAD. **Intensity:** As above. **PPI:** As above	**Symptom:** SIAS, SPS, BDI **Shame:** Experience of Shame Scale	Shame proneness reduced significantly in both the self-help (MD = 7.34, Cohen's *d* = 0.80, 95% CI 3.99–10.69, *p* < 0.001) and guided ICBT groups (MD = 9.97, Cohen's *d* = 0.88, 95% CI 5.36–14.56, *p* < 0.001) after treatment, with no significant differences between the two intervention groups. Modules include relaxation training (*r* = −0.24, *p* = 0.03), problem solving (*r* = −0.25, *p* = 0.03), and exposure (*r* = −0.36, *p* = 0.001) contributed to the reduction of shame level.	2.1 = 2 2.2 = 1 2.3 = 0 2.4 = 0 2.5 = 0
Chen et al. (2020), mainland China	Non-RCT; anxiety; *N* = 255 (IG = 183, CG = 72)	**Study aim:** To identify demographic and clinical factors associated with treatment adherence and outcomes in ICBT for social anxiety, and to explore whether low-intensity therapist support results in improved treatment adherence or outcomes Comparator: wait-list control. **Intensity:** As above. **PPI:** As above	**Acceptability:** Drop-out defined as the failure to complete outcome assessments based on the intent-to-treat sample size of the intervention group and participants who were allocated to the intervention but never started the intervention; adherence defined as the number of completed modules of the intervention, ranging from 0 to 8. **Symptom:** SPS and SIAS.	The drop-out rate was 60% in the self-guided group and 54% in the therapist-guided group, with no significant between group differences (OR 0.802, *p* = 0.43). Participants completed 4.1 modules on average with no between group differences (*t* = 1.04, *p* = 0.03). Age (*B* = 0.17, s.e. = 0.04, *p* = 0.008) and SAD diagnosis (*B* = 0.16) were predictors of adherence, with older participants and participants with a diagnosis of SAD tended to complete more modules. Gender (*B* = −0.20, s.e. = 0.18, *p* = 0.04) and number of completed modules (*B* = 0.24, s.e. = 0.03, *p* = 0.01) as significant predictors of residual gain score for SIAS, with participants who identified as female and those who competed more modules reporting greater improvement in SIAS scores.	3.1 = 0 3.2 = 1 3.3 = 0 3.4 = 1 3.5 = 0
Yeung et al. (2018), mainland China	RCT; depression; *N* = 75 (IG = 37, CG = 38)	**Study aim:** To examine the feasibility, safety, and effectiveness of using an online computerised cognitive behavioural therapy (CBT) for treating Chinese patients with depression. **Intensity:** 1 module each week for 5 weeks. **PPI:** No.	**Symptom**s: CES-D, **Acceptability and usability:** satisfaction survey	The improvement in CES-D of the intervention group was significantly greater than the control group (*t* = 2.37, *p* = 0.02). Acceptability and usability of MoodGYM was as follows: ‘The content was easy to understand’ (37%); ‘The wording was easy to understand’ (35%); ‘Easy to use’ (65%); ‘Too long (64%)’, ‘Useful in helping to understand depression’ (100%); ‘Useful in improving mood’ (63%); ‘Would recommend to friends or family’ (36%); and ‘Interested in more online training’ (66%). No serious adverse events were reported.	2.1 = 1 2.2 = 0 2.3 = 1 2.4 = 0 2.5 = 1
Ren et al. (2016), mainland China	RCT; depression; *N* = 62 (IG = 47, CG = 15)	**Study aim:** To test the effectiveness of an internet-based intervention programme (MoodGYM) among college students with depression, and to explore the mediation mechanism of cognitive distortion and interpretation bias. **Intensity:** Complete 1 module per 3 days for 3 weeks. **PPI:** No.	**Symptoms:** CES-D, PHQ-9	There was a significantly greater reduction in the intervention group in depressive symptoms compared to the control group post-treatment assessment (CES-D, *F* = 7.21, *p* = 0.01; PHQ-9, *F* = 5.14, *p* = 0.028). No significant medication effect was observed.	2.1 = 1 2.2 = 1 2.3 = 0 2.4 = 0 2.5 = 2
Patel et al. (2017), mainland China	Qualitative study; depression; *N* = 20	**Study aim:** To explore cultural adaptation of the CATCH-IT for use in mainland China. **Intensity:** 2-months, 2 modules per week. **PPI:** No.	**Acceptability:** standardised questionnaire for expert panel (a questionnaire about cultural adaptation and implementation in mainland China); feedback survey for students (a questionnaire to identify facilitators, barriers, and suggestions for improvement of the website)	CATCH-IT was an acceptable internet-based intervention to prevent depression for adolescents. A translation from English to Mandarin Chinese and cultural adaptation was suggested by both expert and student feedback. More Chinese cultural relevant themes and more interactive features and less text on the website were recommended.	1.1 = 1 1.2 = 1 1.3 = 0 1.4 = 0 1.5 = 0
Wang et al. (2013b), mainland China	RCT; trauma; *N* = 183 (IG: urban = 46, rural = 49; CG: urban = 44, rural = 44)	**Study aim:** To investigate the efficacy of an internet-based trauma intervention (named CMTR). **Intensity:** 1-month, self-paced for urban participants, 5 sessions, each session for at least 0.5 h per 5 days for rural participants. **PPI:** No	**Symptom:** PDS, SCL-D, CSE, PCC **Function:** SFI	After 1-month treatment, the urban subgroup scored significantly lower on PDS (*F* = 4.29, *p* = 0.04), PCC (*F* = 8.50, *p* = 0.005), SFI (*F* = 5.08, *p* = 0.03), and SCL-D (*F* = 4.32, *p* = 0.04) than wait list group, while the between group differences in the rural sub-group was significant only on PDS (*F* = 6.86, *p* = 0.01). The reduction was sustained over a 3-month follow-up in both urban sample and rural samples.	2.1 = 1 2.2 = 1 2.3 = 0 2.4 = 0 2.5 = 0
Wang et al. (2013a), mainland China	RCT; trauma; *N* = 103 (IG = 50, CG = 53)	**Study aim:** To examine the user dropouts at different stages of CMTR, and to identify the factors affecting the usage of the programme. **Intensity:** 1-month, self-paced. **PPI:** No.	**Symptoms:** PDS, DTQ, SAQ **Acceptability:** Satisfaction questionnaire	Among 103 participants, 61 (59.2%) used the website, and 42 (40.8%) dropped out before the treatment. The users' visiting days at the website were positively correlated with the SAQ family disapproval scores (*β* = 0. 31, *p* < 0.05), and the number of pages visited was positively correlated with the SAQ general disapproval scores (*β* = 0. 31, *p* < 0.05).	2.1 = 1 2.2 = 1 2.3 = 0 2.4 = 0 2.5 = 0
Wang et al. (2016), mainland China	Secondary analysis of Wang et al. (2013b); trauma; *N* = 146	**Study aim:** To examine the usability of CMTR in rural and urban participants, and to investigate the predictors of programme use and the impact of programme usage on study outcomes. **Intensity:** 1-month, self-paced. **PPI:** No	**Acceptability and usability:** Reflected by programme use: total number of days visiting CMTR, total number of CMTR webpages completed, number of modules visited, number and proportion of webpages completed in each module, number of modules visited per day, number and proportion of webpages completed in each module on the first day. **Symptoms:** PDS, SCL-D **Functional outcomes:** SFI, CSS, CSE	**Programme use:** Rural participants had a significant larger total number of visiting days (*F* = 40.50, *p* < 0.001) and visited more programme modules in 1 month than urban participants (χ^2^ = 73.67, *p* < 0.001). **Predictors and programme use:** Total number of visiting days was positively correlated with CSS at pre-test (*r* = 0.22, *p* = 0.009), and the total number of completed webpages was positively correlated with SFI at pre-test (*r* = 0.19, *p* = 0.02). **Programme use and outcomes change:** In general, use of the triggers and self-talk modules showed a consistent positive association with improvement in PDS, SCL-D, SFI, and CSE.	2.1 = 1 2.2 = 1 2.3 = 0 2.4 = 0 2.5 = 0
Li et al. (2017), mainland China	RCT; ADHD; *N* = 34 (IG = 17, CG = 17)	**Study aim:** To evaluate the effects of the internet-based executive function training on the core symptoms and executive function in children with ADHD. **Intensity:** 12-week, 5 sessions per week, each session for 24 min. **PPI:** No.	**Symptoms:** ADHD RS-IV. **Executive function:** self-developed cognitive tests, BRIEF	A significant decrease of ADHD symptoms was found in both intervention (*F* = 15.624, *p* < 0.01) and control group (*F* = 16.830, *p* < 0.01) post-treatment with no significant group differences (*F* = 0.287, *P* > 0.05). The intervention group showed significant improvement in speech working memory (*F* = 5.044, *p* < 0.05), spatial working memory (*F* = 8.319, *p* < 0.01), and rapidly visual information search scores (*F* = 6.160, *p* < 0.05) post-treatment, but no between group differences were found.	2.1 = 2 2.2 = 0 2.3 = 1 2.4 = 2 2.5 = 2
*Smartphone app*					
Sun et al. (2019), mainland China	RCT; anxiety; *N* = 38 (IG = 19, CG = 19)	**Study aim:** To evaluate the effect of the cognitive bias modification smartphone app on social anxiety, and to examine the mediators of change in social anxiety through longitudinal data. **Intensity:** 4-weeks, twice per week. **PPI:** No.	**Symptoms:** LSAS, STAI	Social anxiety reduced significantly in the intervention group compared to controls post-test (*t* = 5.20 *p* = 0.03), and the post-test score did not differ significantly with follow-up. No significant mediation effect was indicated.	2.1 = 2 2.2 = 1 2.3 = 1 2.4 = 0 2.5 = 1
Yang et al. (2017), mainland China	RCT; anxiety; *N* = 76 (IG1 = 20, IG2 = 20, IG3 = 16, CG = 20)	**Study aim:** To test the effectiveness of three different types of training programmes (CBM-A, CBM-I, AIM) administered via a smartphone app in people with social anxiety. **Intensity:** Single training session for 40 min. **PPI:** No.	**Cognitive outcomes:** Attention Bias Modification Assessment Task, WSAP **Symptom:** LSAS, STAI	The effects of CBM-A, CBM-I, AIM *v*. control condition for attention yielded no significant differences in attention bias scores. Only the CBM-I group showed significantly less threat interpretation and more benign interpretation than the control condition on interpretation bias scores (*d* = 0.55, *p* < 0.001).	2.1 = 0 2.2 = 1 2.3 = 1 2.4 = 0 2.5 = 1
Zhu *et al*. (2018b), mainland China	RCT; SUD; *N* = 40 (IG = 20, CG = 20)	**Study aim:** To test the efficacy of a smartphone app on cognitive impairments, eliminate drug-related attention bias, and attenuate risk decision-making behaviours in people with MUD. **Intensity:** 4-weeks, 5 sessions per week (60 min per session, 20 sessions in total). **PPI:** No.	**Cognitive outcomes:** the CogState Battery, DDT, IGT, BART	Significant improvements were observed in the CogState tests and risk decision-making tasks in the intervention group; no significant changes evident in the control group. No patients reported any discomfort during the whole training session.	2.1 = 2 2.2 = 1 2.3 = 1 2.4 = 1 2.5 = 1
Liang et al. (2018a), mainland China	RCT; SUD; *N* = 75 (IG = 50, CG = 25)	**Study aim:** To test the feasibility and outcomes of a smartphone app (S-Health) among individuals using illicit drugs. **Intensity:** Completing a daily survey and receiving 2 health messages every day for 4 weeks. **PPI:** Yes. The app was developed according to participant's feedback from a series of focus groups.	**Drug-related outcomes:** Urine test, timeline follow-back survey, addiction severity index **Usability:** the usability items	At the end of the 1-month study period, no significant difference was observed between the intervention group and the control group; a lower percentage of participants had a positive urine test (26.2% *v*. 50%, *p* = 0.06). The number of days using drugs in the past week was significantly lower among participants in the intervention group (mean = 0.71, s.d. = 1.87) relative to the control group (0.71 *v*. 2.20, *p* < 0.05). Most of the participants in the intervention group strongly agreed or agreed that the survey questions were easy to understand (55.3%) and the smartphone screens were easy to use (72.3%), and preferred answering questions on the smartphone app (46.8%) relative to in-person interview assessment (36.2%).	2.1 = 2 2.2 = 1 2.3 = 1 2.4 = 0 2.5 = 0
Han et al. (2018), mainland China	Secondary analysis of Liang et al. (2018a ); SUD; *N* = 75 (IG = 50, CG = 25)	**Study aim:** To examine the feasibility of an EMA smartphone app by testing the concordance of drug use assessed by the app, urine testing, and a LET assessment. **Intensity:** Same as Liang et al. (2018a, 2018b). **PPI:** Same as Liang et al. (2018a ).	**Drug-related outcomes:** EMA, LET, urine test **Acceptability:** Post-Intervention Acceptability Survey	The study showed poor agreement between the EMA data and the LET and the urine test. In the 4-week period, the agreement between the EMA and the LET was 66.7, 79.2, 72.4, and 85.8%, and the agreement between the EMA and the urine test was 51.2, 65.1, 61.9, and 71.5%, respectively. Acceptability was low; 46% of participants preferred face-to-face interviews rather than using the app.	2.1 = 2 2.2 = 1 2.3 = 1 2.4 = 0 2.5 = 0
Schulte et al. (2016), mainland China, Taiwan, the USA	Qualitative study; SUD; *N* = 72 (mainland China participants n = 18)	**Study aim:** To obtain heroin-dependent patients’ perspectives on acceptance and potential adoption for a smartphone application (‘S-Health’) in mainland China, Taiwan, and the USA. **Intensity:** Same as above. **PPI:** Same as above.	**Acceptability:** Focus groups	Participants showed general acceptance of the app. Regarding compatibility, participants reported that the app could help support recovery needs and goals, but its utility during strong craving was questioned. In terms of complexity, participants mentioned smartphone access and familiarity, individualisation of content, and privacy and security.	1.1 = 1 1.2 = 1 1.3 = 1 1.4 = 1 1.5 = 1
Xu et al. (2021), mainland China	RCT; SUD; *N* = 40 (IG = 20, CG = 20)	**Study aim:** To test the feasibility and preliminary efficacy of the programme (CAREs) in SUD in a community-based setting. **Intensity:** Required to log in at least once a week. **PPI:** No.	**Feasibility:** The overall proportion and frequency of CAREs features used **Drug-related outcomes:** Urine drug screen, ASI	**Feasibility:** 100% participants accessed the assessment feature followed by education (75%); and the most frequently used feature was education (mean total time = 63.3). Coping skills and support functions showed low levels of use. **Drug-related outcomes:** Significant lower percentage of drug-positive urine samples in intervention group than control group. No significant between differences were found on other outcome measures.	2.1 = 1 2.2 = 1 2.3 = 1 2.4 = 0 2.5 = 1
An et al. (2017), mainland China	Case series; ASD; *N* = 10	**Study aim:** To evaluate the effectiveness of training for making requests using Yuudee in ten minimally verbal children with ASD. **Intensity:** 5-weeks, one or two sessions per week (30 min per session, eight sessions in total). **PPI:** No.	**Training effectiveness:** Prompted response	The accuracy rate of a given phase was calculated for each child who achieved three consecutive unprompted successful responses in the phase. Seven children achieved at least 50% accuracy in at least two of the five phases. The other three children achieved at least 50% accuracy in only one phase. Two children achieved at least 50% accuracy in all of the phases in which they were trained.	4.1 = 1 4.2 = 0 4.3 = 1 4.4 = 1 4.5 = 1
Zhang et al. (2019), mainland China	Case series; ASD; *N* = 3	**Study aim:** To evaluate efficacy of the app on improving facial expression recognition and emotion understanding abilities in children with ASD. **Intensity:** 8-weeks, one session per week (25 min for each session). **PPI:** No.	**Training effectiveness:** Accuracy of the training test	All of the three participants achieved an improvement post-treatment compared to baseline in the distinguishing phase (percentage of the mean score increased 21.9, 44, 35.1%), and achieved sustainable above 80 during the maintenance phase. For the Understanding phase, two participants achieved an improvement after treatment (51% and 58%), and one participant's baseline score is above 80 therefore received no treatment, and all participants remained above 80 in maintenance phase.	4.1 = 1 4.2 = 0 4.3 = 0 4.4 = 1 4.5 = 1
Sun et al. (2021), mainland China	RCT; perinatal depression; *N* = 168 (IG = 84, CG = 84)	**Study aim:** To evaluate the effectiveness of a smartphone-based mindfulness training on perinatal depression. **Intensity:** 8-weeks, one session per week (30 min per session, eight sessions in total). **PPI:** No.	**Symptoms**: EPDS, GAD-7, Perceived Stress Scale, Positive and Negative Affect Schedule, Pittsburgh Sleep Quality Index, Fatigue Severity Scale, Prospective and Retrospective Memory Questionnaire, the Wijma Delivery Expectancy Questionnaire	A significant improvement of depression (χ^2^_4_ = 16.2, *p* = 0.003), anxiety (χ_4_^2^ = 13.1, *p* = 0.01), and positive affect (χ_4_^2^ = 8.4, *p* = 0.04) was observed in the intervention group compared to control participants. There were medium between-group effect sizes on depression (Cohen's *d* = 0.47) and positive affect (Cohen's *d* = −0.49) at postintervention, and on anxiety (Cohen's *d* = 0.46) in late pregnancy.	2.1 = 1 2.2 = 1 2.3 = 0 2.4 = 1 2.5 = 0
*Text messaging*					
Chen et al. (2010), mainland China	Case series; suicide; *N* = 15	**Study aim:** To test the feasibility and acceptability of mobile phone text messages contacts after discharge in people with suicide attempts. **Intensity:** 4-weeks, once per week. **PPI:** No	**Acceptability, usability:** Interview	None of the participants responded to the text message. All participants read all four messages. Twelve participants felt it was acceptable and were willing to use it for a longer period. Three participants felt it was not helpful and refused to receive further messages.	1.1 = 1 1.2 = 1 1.3 = 0 1.4 = 0 1.5 = 0
Xu et al. (2019), mainland China	RCT; schizophrenia; *N* = 278 (IG = 139, CG = 139)	**Study aim:** To test the effectiveness of lay health supporters (family members or community volunteers) aided by a simple text messaging intervention for increasing medication adherence, and improving symptoms and functioning in people with schizophrenia living in a rural community. **Intensity:** 6-months, 2 messages per day (9:00 am and 7:00 pm). **PPI:** No.	**Medication adherence:** Score of adherence to antipsychotic medications (the proportion of dosages taken over the past 30 days), BARS, DAI-10, medication refill records. **Symptoms:** CGI for schizophrenia. **Functional outcomes:** WHODAS 2.0	The intervention group showed significant improvements in medication adherence (0.48 *v*. 0.61, *p* = 0.013) and a substantial reduction in risk of relapse (RR = 0.63, 95% CI 0.42–0.92) and re-hospitalisation (RR = 0.36, 95% CI 0.17–0.73) compared to the control group.	2.1 = 1 2.2 = 1 2.3 = 1 2.4 = 1 2.5 = 1
Cai et al. (2020), mainland China	Approximated stepped-wedge RCT; schizophrenia; *N* = 277 (this is the extended implementation phase of Xu et al. (2019))	**Study aim:** To test the effectiveness of the text messaging programme on improving medication adherence, functioning, and symptoms in people with schizophrenia by an extended implementation of the intervention after its initial phase. **Intensity:** Same as Xu et al. (2019). **PPI:** Same as Xu et al. (2019).	**Medication adherence:** Score of adherence to antipsychotic medications (the proportion of dosages taken over the past 30 days), BARS, DAI-10, medication refill records. **Symptoms:** CGI for schizophrenia. **Functional outcomes:** WHODAS 2.0	A significant improvement in medication adherence (adjusted mean difference 0.11, 95% CI 0.04–0.19; *p* = 0.004), symptoms (adjusted mean difference −0.26, 95% CI −0.50 to −0.02; *p* = 0.04; Cohen's d = 0.20) and a reduction in rehospitalisation (0.37, 95% CI 0.18–0.76; *p* = 0.007; number-needed-to-treat 8.05, 95% CI 4.61–21.41) in the extended intervention period compared to the control period (the initial phase) was found.	2.1 = 1 2.2 = 1 2.3 = 1 2.4 = 1 2.5 = 1
Wang et al. (2017), mainland China	Qualitative study; schizophrenia; *N* = 16 (8 people with schizophrenia and 8 family members)	**Study aim:** To explore the acceptability of the text messaging intervention. **Intensity:** Same as Xu et al. (2019). **PPI:** Same as Xu et al. (2019).	**Feasibility:** Semi-structured interviews	Of the 16 participants, 5 people with schizophrenia and all of the family members were willing to receive messages. Participants did not fully master sending and receiving messages via the mobile phone. Participants reported that the messages were helpful and useful.	1.1 = 1 1.2 = 1 1.3 = 1 1.4 = 1 1.5 = 1
*Social media*					
Zhu *et al*. (2020c), mainland China	RCT; schizophrenia; *N* = 84 (IG = 42, CG = 42)	**Study aim:** To evaluate the effects of a WeChat-based intervention on medication adherence and quality of life in people with schizophrenia. **Intensity:** 6-months, medication reminders 2–4 times a day, education messages once a week. **PPI:** No.	**Medication adherence:** MAQ **Quality of life:** SQLS	**Medication adherence:** A significant between group difference (*F* = 28.087, *p* < 0.001, *η*_p_^2^ = 0.255), within time differences (*F* = 112.871, *p* < 0.001, *η*_p_^2^ = 0.579), and group × time interaction (*F* = 28.726, *p* < 0.001, *η*_p_^2^ = 0.259) were found, with better medication adherence in the WeChat group (month 3, *M* = 1.43, s.d. = 0.55; month 6, *M* = 1.31, s.d. = 0.56) than the control group (month 3, *M* = 2.09, s.d. = 0.62; month 6, *M* = 2.64, s.d. = 0.58) at both 3 and 6 months. **Quality of life:** A significant between group difference (*F* = 29.546, *p* < 0.001, *η*_p_^2^ = 0.265), within time difference (*F* = 259.885, *p* < 0.001, *η*_p_^2^ = 0.760), and group × time interaction (*F* = 102.225, *p* < 0.001, *η*_p_^2^ = 0.555) was found, with lower SQLS total score in the WeChat group (month 3, *M* = 76.87, s.d. = 9.82; month 6, *M* = 74.23, s.d. = 10.21) than the control group (month 3, *M* = 82.03, s.d. = 8.75; month 6, *M* = 103.01, s.d. = 9.83) at both 3 and 6 months.	2.1 = 1 2.2 = 1 2.3 = 1 2.4 = 1 2.5 = 1
Zhang et al. (2021), mainland China	Case series; insomnia; *N* = 194	**Study aim:** To explore the efficacy of a WeChat-based self-guided cognitive behavioural treatment for insomnia on situational insomnia during the COVID-19 pandemic. **Intensity:** 1-week, one session per day, each session lasting 10–15 min. **PPI:** No.	**Symptoms:** PSAS, ISI, HADS	ignificant improvements were found on PSAS total score and cognitive score (*p* = 0.004, *p* < 0.001), ISI total score (*p* < 0.001), and both the HADS anxiety and depression score (*p* < 0.001) post-treatment. Participants completed all seven sessions and showed significantly better outcomes on PSAS total score, somatic score, cognitive score (*p* = 0.003, *p* = 0.014, *p* = 0.009), and HADS anxiety score (*p* = 0.045)	4.1 = 1 4.2 = 1 4.3 = 1 4.4 = 0 4.5 = 1
*Virtual reality*					
Zhang *et al*. (2016a), mainland China	non-RCT; ASD; *N* = 15 (IG1 = 5, IG2 = 5, CG = 5)	**Study aim:** To explore the effectiveness of VR in promoting peer interaction ability in children with high functioning autism. **Intensity:** 4-weeks, weekly for 2 h. **PPI:** No.	**Attention bias:** Accurate rate and reaction time **Symptoms:** ADOS	Participants in experiment group 1 (attention bias modification and peer interaction) performed significantly better on reaction time (*U* = 1.0, *p* = 0.016) and accuracy (*U* = 0.0, *p* = 0.008), ability to pay attention to others (*U* = 0.5, *p* < 0.01), and using language (*U* = 2.0, *p* < 0.05) than experiment group 2 (peer interaction only). Social skills in both experiment groups were significantly higher than the control group after intervention.	3.1 = 0 3.2 = 1 3.3 = 1 3.4 = 0 3.5 = 0
Zhang et al. (2020), mainland China	Case series; anxiety; N = 3	**Study aim:** To explore the efficacy of VR exposure therapy in treating fear of COVID-19. **Intensity:** Repeated exposure with the same stimulus until achieved 50% decrease on self-rated the Subjective Units of Distress Scale. **PPI:** No.	**Symptoms:** HAMA, FCV-19S	A significant decrease on mean score anxiety symptoms (*F* = 31.681, *p* = 0.030) between the pre-treatment (*M* = 23.33, s.d. = 7.02) and post-treatment (*M* = 7.67, s.d. = 2.89). No significant change on COVID-19 phobic symptoms.	4.1 = 1 4.2 = 0 4.3 = 1 4.4 = 1 4.5 = 1
Lyu et al. (2020); mainland China	RCT; major depressive disorder and bipolar disorder depressive episodes; *N* = 64 (IG1 = 23, IG2 = 21, CG = 20)	**Study aim:** To investigate the effect of VR attention training on cognitive function in depression. **Intensity:** 4-weeks, 5 sessions per week, each session lasting 30 min. **PPI:** No.	**Cognitive functions:** MCCB **Symptoms:** HAMD, HAMA	**Cognitive functions:** At post-treatment, VR group showed significant higher **i**nformation processing speed and attention/alertness scores than both CCRT group and the control group (*p* < 0.05), and both the VR group and the CCRT group showed significant higher visual learning scores than the control group (*p* < 0.01). **Symptoms:** HAMD and HAMA decreased significantly after treatment in all of the three conditions, but no group differences were found.	2.1 = 1 2.2 = 1 2.3 = 1 2.4 = 1 2.5 = 1

RCT, randomised controlled trail; IG, intervention group; CG, control group; MCCB, MATRICS Consensus Cognitive Battery; WCST, Wisconsin Card Sort Test; UPSA, UCSD Performance-based Skills Assessment; NOSIE, Nurse's Observation Scale for Inpatient Evaluation; PANSS, Positive and Negative Syndrome Scale; CCRT, Computerised cognitive remediation therapy; BARS, Brief Adherence Rating Scale; DAI, Drug Attitude Inventory; CGI, Clinical Global Impression; WHODAS, WHO Disability Assessment Schedule; LSAS, Liebowitz Social Anxiety Scale; STAI, State-Trait Anxiety Inventory; SUD, substance use disorder; DDT, delay discounting task; IGT, Iowa gambling task; BART, balloon analogue risk task; CCAT, Computerised Cognitive Addiction Therapy; EMA, Ecological Momentary Assessment; LET, life experience timeline; PDS, Posttraumatic Diagnostic Scale; SCL-D, Symptom Checklist -Depression; SFI, Social Functioning Impairment; CSS, Crisis Support Scale; CSE, Coping Self-Efficacy; PCC, Post-traumatic Cognitive Changes; RPM, Raven's Progressive Matrices; CAST, Childhood Autism Spectrum Test; PEP-3, Psychoeducational Profile Third Edition; ABAS-II, Adaptive Behaviour Assessment System – Second Edition; CESD-R, Centre for Epidemiologic Studies Depression Scale – Revised; ITT, intention to treat; SSQ, Simulator Sickness Questionnaire; VQ, Volitional Questionnaire; CES-D, Centre for Epidemiologic Studies Depression Scale; PSP, Personal and Social Performance Scale; SIAS, Social Interaction Anxiety Scale; SPS, Social Phobia Scale; BDI, Beck Depression Inventory; IPROS, Inpatient Psychiatric Rehabilitation Outcome Scale; ASD, autism spectrum disorder; non-RCT, non-randomised controlled trial; ADOS, Autism Diagnostic Observation Schedule; BPRS, Brief Psychiatric Rating Scale; CGI-S, Clinical Global Impression-Severity; ACCT, A battery of computerised cognitive test; PHQ, Patient Health Questionnaire; VAS, Visual Analogue Scale; WHOQOL-BREF, WHO Quality of Life-Brief; SWLS, Satisfaction With Life Scale; TKSS, Taijin Kyofusho Scale; WSAP, Word Sentence Association Paradigm; LSAS, Liebowitz Social Anxiety Scale; STAI, State Trait Anxiety; PDS, Posttraumatic Diagnostic Scale; DTQ, Disclosure of Trauma Questionnaire; SAQ, Social Acknowledgement Questionnaire; CBM-A, cognitive bias modification-attention; CBM-I, cognitive bias modification-interpretation; AIM, attention and interpretation modification; ASI, addiction severity index; MAQ, Medication Adherence Questionnaire; SQLS, Schizophrenia Quality of Life Scale; PSAS, Pre-sleep Arousal Scale; ISI, insomnia severity index; HADS, Hospital Anxiety and Depression Scale; HAMA, Hamilton Anxiety Scale; FCV-19S, Fear of COVID-19 Scale; TAU, treatment as usual; ADHD, attention deficit hyperactivity disorder.

^a^The criteria for each of the MMAT items (Hong et al. 2018) are shown in online Supplementary Table S2.

b Taijin Kyofusho is a culturally bound form of social anxiety, mostly seen in Eastern cultures, where people worry that their behaviours, expressions, or physical characteristics will offend or make others feel uncomfortable (Kleinknecht et al., 1997).

### Characteristics of participants

Selected studies investigated a variety of mental health problems, including schizophrenia (*n* = 12), depression (*n* = 7), anxiety (*n* = 6), substance use disorder (SUD; *n* = 5), autism spectrum disorder (ASD; *n* = 3), trauma (*n* = 3), attention deficit hyperactivity disorder (ADHD; *n* = 1), insomnia (*n* = 1), and suicide prevention (*n* = 1). The total number of people with mental health problems recruited from mainland China was 3112. The mean age of participants with mental health problems ranged from 4.7 to 47.4 years. The total number of female and rural participants was 1676 (54%) and 395 (13%), respectively.

### Overview of the measurement of outcomes

Where studies examined efficacy or effectiveness of DHTs, outcomes included psychiatric symptomatology, cognitive outcomes, functional outcomes, shame, and medication adherence (see Table 1 and online Supplementary Table S3). Where studies evaluated acceptability and usability, survey and interview data, and programme use (e.g. time spent on website) were reported.

### Characteristics of DHTs

A summary of intervention characteristics is demonstrated in Table 1 (see online Supplementary Table S3 for a more detailed summary of studies). In total, 32 DHTs were identified from included studies; of these, six DHTs were adapted from existed tools developed in Western countries (Hu et al., 2021; Kishimoto et al., 2016; Patel et al., 2017; Sit et al., 2020; Wang, Wang, & Maercker, 2013b; Yeung et al., 2018). Only one of the selected studies involved patient and public involvement methods in the DHT development process (Schulte et al., 2016). The length and duration of DHTs varied widely, ranging from one session to an 8-month intervention window.

### Acceptability, usability, and safety of DHTs in the Chinese context

#### Internet-based programmes

Five studies reported acceptability and usability data on internet-based programmes for depression, anxiety, and trauma. In general, most participants deemed the programmes to be helpful and useful. One study reported 100% of participants reported that the programme was useful, while 64% participants felt the programme was too long (Yeung et al., 2018). Older participants (Chen et al., 2020), participants with more severe symptoms (Chen et al., 2020; Wang, Wang, & Maercker, 2013a), and participants with higher functioning levels (Wang, Wang, & Maercker, 2016) showed higher rates of engagement with the programme. One qualitative study (Patel et al., 2017) indicated that substantial cultural adaptation was needed for a programme originally developed in the USA. One study (Yeung et al., 2018) examined safety of an internet-based programme and no severe adverse events were observed. Overall, participants considered internet-based programmes acceptable and helpful, although some negative feedback was expressed (e.g. the content of the programme was too long).

#### Smartphone apps

Five studies evaluated the acceptability and feasibility of two smartphone app-based interventions: one study on a smartphone app for perinatal depression and the other four studies about an app for SUD. One RCT (Sun et al., 2021) reported poor intervention completion rates for an app-based mindfulness training on perinatal depression; 52.3% of participants in the intervention group completed at least 4 weeks of the intervention and only 8% completed the full 8-week intervention programme. For smartphone apps for SUD, participants from one RCT study (Liang, Han, Du, Zhao, & Hser, 2018a) reported that the content was easy to understand in only 55% of cases, although 72% said that the app was easy to use. One focus group study (Schulte et al., 2016) reported that receiving support via the app helped people avoid feeling ashamed of sharing personal information with others compared to attending services in-person. However, another study indicated limited acceptance; 46% of participants preferred in-person services instead of using the app (Han et al., 2018). In summary, the feasibility of app-based interventions for perinatal depression was limited, and contradictory results were found for smartphone app-based programmes for SUD; some participants felt the app was acceptable, while others preferred in-person services.

#### Text messaging

Two studies investigated the acceptability and usability of a text messaging intervention for people who experienced a suicide attempt and people with chronic schizophrenia. In the post-treatment interview, 80% (12/15) of people who experienced a suicide attempt reported that receiving text messages was acceptable and they were willing to receive the messages for a longer period (Chen, Mishara, & Liu, 2010). Another small qualitative study (Wang et al., 2017) found that five out of the eight people with chronic schizophrenia and all of the family members who participated in the interview reported that the text messaging intervention was acceptable. However, not all of the participants and their family members mastered using a mobile phone, mainly due to low literacy level, older age, and cognitive impairment. In summary, these results showed that text messaging interventions are generally acceptable.

### Effects of DHTs on symptoms, neurocognitive, and functional outcomes in the Chinese context

#### Computerised programmes

Ten studies examined computerised programmes in people with a diagnosis of schizophrenia and depression. Eight studies tested the effects of computerised cognitive remediation therapy (CCRT) improving neurocognitive outcomes for people with schizophrenia. Seven RCT studies (Byrne et al., 2013; Hu et al., 2021; Liao, Ding, Wu, Zhou, & Pan, 2016; Tan et al., 2019; Zhang, Xu, Chen, & Chen, 2016b; Zhu et al., 2018a; Zhu et al., 2020b) found computerised programmes improved neurocognitive functioning in schizophrenia patients compared with a control group. Of these, two studies (Byrne et al., 2013; Tan et al., 2019) found improved negative symptom and psychotic symptom scores of the Positive and Negative Syndrome Scale (PANSS) compared with a control group, and two studies (Zhang et al., 2016b; Zhu et al., 2020b) found that the CCRT programme significantly improved functional outcomes compared to controls. Conversely, Byrne, Pan, McCabe, Mellor, and Xu (2015) conducted a non-RCT and found no significant difference in symptoms and neurocognitive outcomes between the intervention group compared with treatment as usual (TAU) plus antipsychotic, but a significant improve in functional outcomes was observed. However, of the eight studies, five were conducted with inpatients and two studies recruited male participants only, which limits the generalisability of study results.

Overall, the efficacy of CCRT programme on improving neurocognitive functioning in schizophrenia patients was demonstrated by all seven RCT studies. These are better effects than trials conducted in Western countries (Kambeitz-Ilankovic et al., 2019), but the efficacy on enhancing psychotic symptoms and functional outcomes remains uncertain. Moreover, computerised programme may help improve neurocognitive functions (Zhu et al., 2020a) and depressive symptoms (Sit et al., 2020) in people with depression; however, the representativeness of findings is limited to people with mild to the moderate levels of depression.

#### Internet-based programmes

Ten studies investigated the effectiveness of internet-based programmes for people with anxiety, depression, trauma, and ADHD. Two RCTs (Lin et al., 2020; Wang et al., 2020) and two non-RCTs (Chen et al., 2020; Kishimoto et al., 2016) tested internet-based cognitive behavioural therapy (ICBT) for social anxiety. All four studies showed ICBT reduced the severity of social anxiety, with up to 42% of participants in one study (compared to 20% of participants in the control group; Lin et al., 2020) showing significant improvements in social anxiety symptoms post-treatment. Lin et al. (2020) also evaluated the effects of internet-based programmes on cultural specific condition, Taijin Kyofusho (TKS), which is a culture-related subtype of social anxiety recognised by both the DSM-5 and the ICD-10. TKS is commonly seen in Eastern cultures manifesting as individuals fear of embarrassing or offending others by behaviours, expressions, or physical characteristics (Kleinknecht, Dinnel, Kleinknecht, Hiruma, & Harada, 1997). Results showed a 44% improvement rate for participants in the intervention group using the TKS Scale. Another RCT study (Wang et al., 2020) explored the effects on reducing shame, a potential mediator of social anxiety. Both guided and unguided internet-based programmes reduced shame proneness compared to the control group. One large-scale non-RCT (Chen et al., 2020, *n* = 255) showed a significant reduction of social anxiety symptoms in the intervention group. However, long-term effects of the interventions were unknown, and drop-out rates of the studies were high, ranging from 41.82% to 59.56%.

Two RCT studies tested the effectiveness of an iCBT programme (the Chinese version MoodGYM) in clinical samples with depression (Ren et al., 2016; Yeung et al., 2018). Significant reductions in depression were reported in both studies post-treatment. However, neither of the studies examined the long-term effects of the programme on depressive symptoms. The results differ from a large-scale pragmatic RCT of MoodGYM conducted in the UK that found no significant improvement of depression compared with controls (Gilbody et al., 2015).

For post-traumatic stress disorder (PTSD), one RCT study (Wang et al., 2013b) evaluated the efficacy in both an urban and rural group. The urban group completed the programme online. The rural group completed the programme at a local counselling centre (due to the lack of access to a computer at home). There was a significant reduction in traumatic symptoms and improved functioning for urban-dwelling participants. For rural participants, there was significant improvement in traumatic symptoms compared to the waitlist control group, but no effect on functioning. For both the urban and rural groups, the effect was sustained at 3-months. However, the drop-out rate in the urban sample was relatively high, with 54% drop-out at post-treatment and 64% at 3-month follow-up in the intervention group.

One RCT study (Li, Liao, Zhou, Wang, and Qian, 2017) tested the efficacy of an online cognitive training programme on improving core clinical symptoms and executive function compared with working memory training in children with ADHD. After 12-weeks of training, both groups showed a significant decrease in clinical symptoms and increased executive function performance, with no between group differences.

In summary, internet-based programmes may have short-term effects on (social) anxiety, depression, trauma, and ADHD, although long-term effects remain uncertain. Improved functional outcomes were evident in studies that included people who had experienced PTSD. High drop-out rates were commonly seen across studies, partially due to the self-help nature of the internet-based programmes.

#### Smartphone apps

Seven studies examined the effect of using a smartphone app for SUD, social anxiety, and ASD. For SUDs, one RCT study (Liang et al., 2018a) assessed a smartphone app on supporting recovery and found the intervention group had a significantly lower number of days using drugs in the past week compared to the control group post-treatment. One RCT study (Xu et al., 2021) tested the efficacy of a smartphone app to support community-based rehabilitation for people with SUDs. Urine drug screens showed a significantly lower percentage of drug positive rates in the intervention group compared with the control group (3.3% *v.* 7.5%). Another small-scale RCT study (*n* = 40; Zhu et al., 2018b) targeting cognitive impairments found the intervention group reported significant improvements in cognitive functions and risk decision-making compared to controls. However, due to the relative small sample size of the three studies and the latter study only including male participants, the generalisability of findings is limited.

Two studies tested smartphone app-based cognitive bias modification programme in people with social anxiety. One RCT study (Sun et al., 2019) showed that the level of social anxiety significantly reduced in the intervention group compared to that in the control group post-treatment and sustained at 1-month follow-up. Another RCT study (Yang et al., 2017) compared three different forms of cognitive bias modification programmes (i.e. attention bias modification, interpretation bias modification, and attention and interpretation bias modification) and found that only the interpretation bias modification group showed a significant reduction in cognitive bias outcomes compared to the control group. However, the generalisability of the results was limited, as both studies recruited university students.

Two case-series studies investigated smartphone app-based interventions on children with ASD. One study (An et al., 2017) evaluated a speech-generating augmentative and alternative communication smartphone app for minimally verbal children with ASD. The training has five phases with each session increasing the degree of difficulty of the task. All participants (*n* = 10) successfully completed at least one phase of the training, and at least 50% accuracy in at least two of the five phases were achieved by seven children, suggesting that the app was feasible for ASD children for speech training. Another study in just three children (Zhang et al., 2019) examined the effects of an app-based intervention on improving facial expression recognition and emotion understanding abilities in children with ASD. The app consisted of seven training domains regarding social communication skills.

Overall, preliminary results of smartphone apps in SUD, social anxiety, and ASD showed feasibility, but clear conclusions regarding efficacy were limited by small samples and a lack of representativeness of study samples.

#### Text messaging

One RCT study (Xu et al., 2019) (*n* = 278) tested a text messaging intervention to increase medication adherence in schizophrenia patients in a resource-limited rural area. Participants in the intervention group and their lay health supporters (i.e. family members or community volunteers) received two text messages daily during a 6-month period. Post-treatment, the intervention group demonstrated significant improvements in medication adherence and a substantial reduction in the risk of relapse and re-hospitalisation compared to the control group. However, the control group received no digital intervention; therefore, treatment effects may be due to non-specific factors. An extended implementation phase was carried out to further explore the effectiveness of the text messaging programme (Cai et al., 2020). In the extended phase, both the intervention group and the control group received the same text messaging intervention. After the 6-month extended intervention, there were further improvements in medication adherence. However, the contribution of the lay health supporters was not examined, which may have resulted in an overestimation of the effects of the text messages.

#### Social media

Two studies examined the effectiveness of a social media-based intervention utilising WeChat, one of the most widely used social media tools in mainland China. One study explored medication adherence in people who had received a diagnosis of schizophrenia, and the other explored insomnia during the COVID-19 pandemic.Zhu et al. (2020c) found a significant increase in medication adherence and quality of life for schizophrenia patients who received the WeChat-based 6-month medication adherence programme compared to controls (who received a telephone check in). However, medication adherence was measured by self-report assessment, which may affect the reliability of reporting due to social desirability and recall bias.

Using a case-series design, Zhang et al. (2021) evaluated the efficacy of a self-guided WeChat-based CBT programme for people who experienced insomnia during the COVID-19 pandemic. After the 1-week intervention, completers (i.e. login time = 7) showed significantly less pro-sleep arousal problems and reported lower insomnia severity scores compared to non-completers (i.e. login time ⩽6). However, due to the lack of control group, the efficacy of the programmes remains uncertain.

#### Virtual reality (VR)

Three studies investigated interventions using VR; one in people with a diagnosis of major depressive disorder or bipolar disorder depressive episodes, one in people with a diagnosis of generalised anxiety disorder specifically related to fear of COVID-19 infection, and one in people with a diagnosis of ASD.

One RCT study (Lyu et al., 2020) evaluated the effectiveness of a VR attention training programme on neurocognitive function compared to CCRT and TAU in people with depressive episode or bipolar disorder. After the 4-week intervention, participants receiving the VR attention training programme showed significantly higher performance on information processing speed and attention/alertness compared with participants received CCRT or no treatment.

One case-series study (Zhang et al., 2020) investigated the effects of VR exposure therapy reducing anxiety due to COVID-19 infection in three participants. Anxiety symptoms decreased significantly at post-treatment; phobic symptoms (i.e. fear of COVID-19 infection) decreased over time but the reduction was statistically non-significant.

One non-RCT (Zhang, Che, Guo, and Lei, 2016a) demonstrated children with ASD significantly increased their ability to pay attention to others and level of language using, and scored higher on social skills compared with the control group post-treatment after receiving a 4-week VR attention bias modification programme. However, the long-term effects were unclear as there was no follow-up period.

In summary, studies primarily tested the efficacy of VR-based interventions in improving neurocognitive function in depression, social skills in ASD, and anxiety. Although feasibility and preliminary efficacy were demonstrated, more rigorous and longitudinal studies with larger sample sizes are needed to evaluate the efficacy of VR interventions.

### Methodological quality

Results of the MMAT quality ratings are shown in Table 1. The methodology of many studies was poorly described and varied in quality across studies. High drop-out rates (i.e. above 20%) were seen in a quarter of the studies, ranging from 27% to 60%, which resulted in incomplete outcome data in these studies. Nearly one-third of included RCTs did not adequately report randomisation procedures, and less than half of RCTs had outcome assessors who were blind to the intervention provided. Most non-randomised controlled studies and case-series studies were poorly representative of the target population.

## Discussion

We systematically and critically appraised current evidence evaluating the efficacy, acceptability, usability, and safety of DHTs across a broad range of mental health problems in China. We identified 39 studies published from 2009 to 2021 which examined 32 DHTs across a range of mental health problems. Of note, most studies were published in the past 5 years, indicating that digital mental health is receiving significant attention and is a fast-growing area of research in China. A quarter of the studies (11/39) were published in Chinese, which demonstrates that a significant number of studies would have been missed if a comprehensive search in Chinese language databases was not conducted; this is indeed a strength of the current study. Compared with papers published in English, Chinese language studies were predominantly RCTs (9/11) and mainly assessed cognitive training interventions (e.g. CCRT).

### Comparison with the broader digital mental health filed

The Chinese studies targeted more often on schizophrenia (12 out of 39 included studies) and SUD (5/39) compared with the field in the Western countries (Miralles et al., 2020) and LMICs (Carter et al., 2021; Fu et al., 2020) where depression was the most studied mental health problem. The different emphasis may reflect how the Chinese mental healthcare system is primarily focused on psychosis and the effort of the government initiated project to manage severe mental health problems (i.e. the ‘686’ programme; Liu et al., 2011) and the extreme limited access to treatment for people with SUD (Liang et al., 2018a). Another explanation may be the researcher deemed digital intervention as an adjunct treatment to medication, as most of the studies focused on neurocognitive training and social skills training, which are domains where medication is less effective (Choi, Wykes, & Kurtz, 2013; Erhart, Marder, & Carpenter, 2006; Kirkpatrick, Fenton, Carpenter, & Marder, 2006). However, some types of digital mental health tools that have been studied in schizophrenia in high-income countries, such as smartphone app-based treatment, symptom monitoring, and self-management tools (Firth & Torous, 2015; Rus-Calafell & Schneider, 2020), have not been developed and tested in China. Unlike the West, cultural-specific conditions have been studied, such as TKS, as well as the target of shame in social anxiety disorders. Although studies included in this review covered a range of prevalent mental health problems in China, there are notable gaps in the development of DHTs for some mental health problems (e.g. bipolar disorder). Moreover, studies disproportionately focused on adult population, while child and adolescent mental health problems received less attention.

Computerised programmes, internet-based programmes, and smartphone apps were the most studied DHTs in this review. This might be attributed to higher access and usage of such technologies compared with other DHTs (e.g. VR). For example, the prevalence of smartphones in China is high, and a significant expansion of the smartphone app market has been witnessed in recent years (Chou et al., 2019; Yin et al., 2020). However, rural settings have fewer internet and smartphone users compared with those living in an urban setting (China Internet Network Information Centre, 2021). Therefore, text messaging interventions might be more feasible in rural or remote settings at this stage (Xu et al., 2019). Additionally, as fewer studies have been conducted in rural areas, the feasibility and effectiveness of DHTs in such settings are poorly understood. Furthermore, similar to Western countries, the efficacy of the majority of apps available on commercial app stores has not been tested using rigorous clinical trial methodology (Torous et al., 2019; Yin et al., 2020). Moreover, some commonly used DHTs have not been tested in China so far, such as wearables.

The second difference is the lack of using co-production approach on DHT development, with co-production was only reported in one included study (Schulte et al., 2016). Systematic co-production involving both mental health professionals and people with mental health problems is considered critical to optimise acceptability (Bucci, Schwannauer, & Berry, 2019). Acceptability and usability were evaluated only in internet-based programmes, smartphone apps, and text messaging interventions. In general, DHTs were acceptable and usable among Chinese people with mental health problems, although contradictory results were reported, especially for smartphone apps for SUDs, indicating that refinement in design and development is necessary to promote acceptability and usability. Co-production has been increasingly implemented in Western countries; however, in China, this approach is primarily utilised in policy making and governance (Li, Hu, Liu, & Fang, 2019; Miao, Schwarz, & Schwarz, 2021) and is relatively new to healthcare research. Future studies should involve co-production in the development and testing process of DHTs to increase engagement and meet service users’ needs (Bucci et al., 2019).

Finally, the methodological quality of the Chinese studies was less rigorous, which may increase the risk of a type 1 statistical error and might be the reason why most of the efficacy studies were positive. For example, all of the included CCRT studies in schizophrenia showed positive effects on improving neurocognitive and functional outcomes, when previous systematic reviews indicated only small to moderate effects and many trials reported negative results (Kambeitz-Ilankovic et al., 2019; Vita et al., 2021). The current review indicated DHTs may play a role in improving symptom, neurocognitive, and functional outcomes in mental health problems in the Chinese population. However, due to the diversity of the studies, no definitive conclusions can be drawn about the effectiveness of identified DHTs. More rigorously designed RCT studies with sufficiently powered samples and long-term follow up are needed to further investigate the effectiveness of DHTs for mental health problems in China and their sustained effects.

### Strengths and limitations

To our knowledge, this is the first review that conducted a comprehensive search in Chinese language databases and included studies published in the Chinese language in the field of digital mental health. Therefore, the cross-cultural generalisability of our findings is robust. Another strength is the inclusion of both quantitative and qualitative design studies. There are also some limitations of the current review. First, differences in outcome assessment, intervention, and comparator conditions precluded meta-analysis. Second, almost all of the included studies reported positive findings, which may suggest publication bias of the review. Finally, there was considerable variation in methodological quality. Nearly half of the RCTs did not report adequate randomisation procedures. Most studies had small sample sizes and were underpowered to detect significant differences, should differences be present. Thus, it is not yet possible to make definitive conclusions about the effectiveness of identified DHTs for mental health problems in China based on the current state of evidence.

### Future research

Future research using larger and more rigorously designed RCTs to evaluate the effectiveness of DHTs is needed, ideally examining a broader range of mental health problems. More large-scale studies with long-term follow-up periods are needed to understand the sustained effects of DHTs. As one of the major advantages of DHTs is its potential to benefit people living in rural/remote settings, more studies in rural areas are needed. Considering most mental health problems develop during adolescence (Kessler et al., 2007), more attention is needed in this group to understand how young people may benefit from DHTs. Finally, in order to ensure DHTs meet the needs of the end-user, and to maximise the likelihood of engagement, the process of designing DHTs should adopt a systematic co-production approach that involve all stakeholders.

## Supplementary material

For supplementary material accompanying this paper visit https://doi.org/10.1017/S0033291721003731. click here to view supplementary material
