# Supplementary material for: Digital mental health in China: a systematic review
Source: Psychol Med. 2021 Sep 28;51(15):2552–70. doi: 10.1017/S0033291721003731 (PMC8579156; doi:10.1017/S0033291721003731)
Supplement: Supplementary file 1 [file S0033291721003731sup.zip › S0033291721003731sup001.docx]

**Supplementary Table 1.** Database search strategy

Search terms in English:

(“serious mental illness” OR “serious and persistent mental illness” OR “severe mental illness” OR “mental illness” OR “mental health” OR “mental disorder” OR “psychotic disorders” OR “psychosis” OR “schizophrenia” OR “schizoaffective disorder” OR “affective disorder” OR “mood disorders” OR “bipolar disorder” OR “hypomania” OR “mania” OR “manic depression” OR “manic-depression” OR “cyclothymic disorder” OR “major depressive disorder” OR “depression” OR “depressive disorder” OR “dysthymic disorder” OR “seasonal affective disorder” OR “postnatal depression” OR “postpartum depression” OR “anxiety” OR “generalised anxiety disorder” OR “social anxiety” OR “obsessive-compulsive disorder” OR “OCD” OR “panic disorder” OR “phobia” OR “phobic disorder” OR “post-traumatic stress disorder” OR “ptsd” OR “trauma” OR “stress disorder” OR “personality disorder” OR “body dysmorphic disorders” OR “eating disorders” OR “anorexia nervosa” OR “binge-eating disorder” OR “bulimia nervosa” OR “attention deficit hyperactivity disorder” OR “ADHD” OR “alcohol addiction” OR “alcoholism” OR “ASD” OR “Asperger syndrome” OR “autism spectrum disorders” OR “autistic disorder” OR “conduct disorder” OR “tic disorders” OR “Tourette syndrome” OR “substance related disorders” OR “drug abuse” OR “heroin dependence” OR “self-harm” OR “self-injurious behavio?r” OR “sleep disorders” OR “insomnia” OR “suicide” OR “suicide identification” OR “suicide prevention” OR “Mental Disorders”[Mesh] OR “Anxiety Disorders”[Mesh] OR “Stress Disorders, Traumatic”[Mesh] OR “Mood Disorders”[Mesh] OR “Depressive Disorder”[Mesh] OR “Psychotic Disorders”[Mesh] OR “Adjustment disorders”[Mesh] OR “alcohol-related disorders”[Mesh] OR “amphetamine-related disorders”[Mesh] OR “anorexia nervosa”[Mesh] OR “anxiety”[Mesh] OR “attention deficit disorder with hyperactivity”[Mesh] OR “Autism spectrum disorder”[Mesh] OR “binge-eating disorder”[Mesh] OR “bipolar disorder”[Mesh] OR “Bulimia nervosa”[Mesh] OR “cocaine-related disorders”[Mesh] OR “depression”[Mesh] OR “depression, postpartum”[Mesh] OR “depressive disorder”[Mesh] OR “depressive disorder, major”[Mesh] OR “Heroin dependence”[Mesh] OR “marijuana abuse”[Mesh] OR “opioid-related disorders”[Mesh] OR “phobia, social”[Mesh] OR “schizophrenia”[Mesh] OR “stress disorders, post-traumatic”[Mesh] OR “stress, psychological”[Mesh] OR “substance-related disorders”[Mesh] OR “suicidal ideation”[Mesh] OR “suicide”[Mesh])

AND

(“telemedicine” OR “telepsychiatry” OR “telehealth” OR “telecare” OR “telemental health” OR “eHealth” OR “electronic health” OR “uHealth” OR “ubiquitous health” OR “mHealth” OR “mobile health” OR “connected Health” OR “online intervention” OR “internet-based intervention” OR “internet health” OR “internet technology” OR “web-based intervention” OR “social media” OR “social network” OR “Facebook” OR “Twitter” OR “Weibo” OR “microblog” OR “WeChat” OR “mobile technology” OR “mobile devices” OR “mobile health technologies” OR “mobile phone” OR “cellular phone” OR “cell phone” OR “smartphone” OR “smartphone application” OR “smartphone app” OR “smartphone technology” OR “app” OR “mobile app” OR “mobile application” OR “text message” OR “SMS” OR “short message service” OR “text messaging” OR “artificial intelligence” OR “digital health” OR “digital medicine system” OR “wearable device” OR “virtual reality” OR “virtual reality intervention” OR “machine learning” OR “computeri?ed” OR “computer-assisted therapy” OR “Therapy, Computer-Assisted” [Mesh] OR “Telemedicine”[Mesh] OR “Internet-based Intervention”[Mesh] OR “Social Media”[Mesh] OR “Cellular Phone”[Mesh] OR “cell phone”[Mesh] OR “Text Messaging”[Mesh] OR “Artificial Intelligence”[Mesh] OR “Therapy, Computer-Assisted”[Mesh] OR “Medical Informatics”[Mesh] OR “mobile application”[Mesh] OR “smartphone”[Mesh] OR “social media”[Mesh] OR “text messaging”[Mesh] OR “virtual reality exposure therapy”[Mesh])

AND

(“China” OR “Chinese” OR “China”[Mesh])

Search terms in Chinese:

SU= (严重精神疾病 OR 严重和持续性精神疾病 OR 严重精神障碍 OR 精神疾病 OR 精神卫生 OR 精神障碍 OR 精神病性障碍 OR 精神病 OR 精神分裂症 OR 分裂情感性障碍 OR 情感障碍 OR 情绪障碍 OR 双相情感障碍 OR 躁狂 OR 轻躁狂 OR 躁狂抑郁症 OR 躁抑症 OR 环性心境障碍 OR 重度抑郁障碍 OR 抑郁症 OR 恶劣心境障碍 OR 季节性情感障碍 OR 产后抑郁症 OR 焦虑症 OR 广义焦虑症 OR 社交焦虑症 OR 强迫症 OR 恐怖症 OR 恐惧症 OR 创伤后应激障碍 OR ptsd OR 创伤 OR 应激障碍 OR 人格障碍 OR 身体畸形恐惧症 OR 进食障碍 OR 神经性厌食症 OR 神经性贪食症 OR 注意缺陷及多动障碍 OR ADHD OR 酒精成瘾 OR 酒精使用障碍 OR 孤独症 OR 阿斯伯格综合症 OR 孤独谱系障碍 OR 自闭症谱系障碍 OR 自闭症 OR 品行障碍 OR 抽动障碍 OR Tourette综合征 OR 物质相关性障碍 OR 物质使用障碍 OR 药物滥用 OR 海洛因依赖 OR 甲基苯丙胺使用障碍 OR 自伤 OR 自伤行为 OR 睡眠障碍 OR 失眠 OR 自杀 OR 自杀识别 OR 预防自杀 )

AND

SU= (远程医疗 OR 远程精神病学 OR 远程医疗 OR eHealth OR uHealth OR mHealth OR 移动医疗 OR 数字医疗 OR 基于互联网的干预 OR 互联网医疗 OR 互联网技术 OR 基于网络的干预 OR 社交媒体 OR 社交网络 OR 微博 OR 微信 OR 移动技术 OR 移动设备 OR 移动健康技术 OR 手机 OR 智能手机 OR 应用程序 OR 智能手机应用程序 OR 智能手机技术 OR app OR 移动应用程序 OR SMS OR 短信 OR 人工智能 OR 可穿戴设备 OR 虚拟现实 OR 虚拟现实干预 OR 机器学习 OR 计算机化 OR 计算机辅助治疗)
